# Supplementary material for: Osteopontin binds ICOSL promoting tumor metastasis
Source: Commun Biol. 2020 Oct 26;3:615. doi: 10.1038/s42003-020-01333-1 (PMC7588454; doi:10.1038/s42003-020-01333-1)
Supplement: Supplementary file 1 — Supplementary Information [file 42003_2020_1333_MOESM1_ESM.pdf]

Supplemental item titles and legends

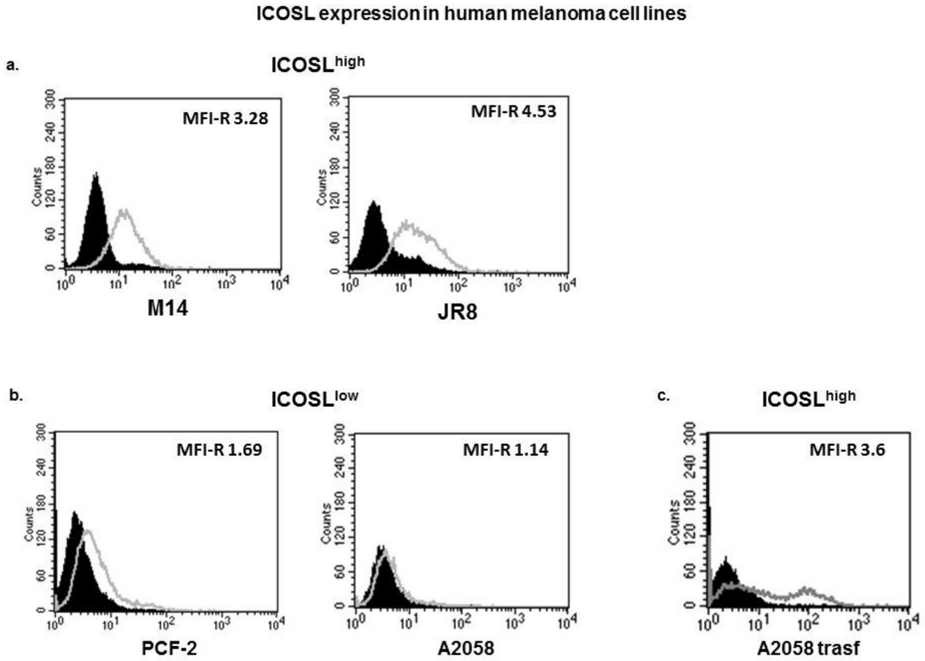

Supplementary Figure 1. ICOSL expression in human melanoma cell lines. a ICOSL expression was assessed by flow cytometry. MFI-ratio >2 identified HIGH expressing cells (**a**- M14, JR8) while MFI-ratio <2 identified LOW expressing cells (**b**- PCF-2 and A2058). **c** ICOSL expression in A2058 cell line, after ICOSL transfection (MFI-R).

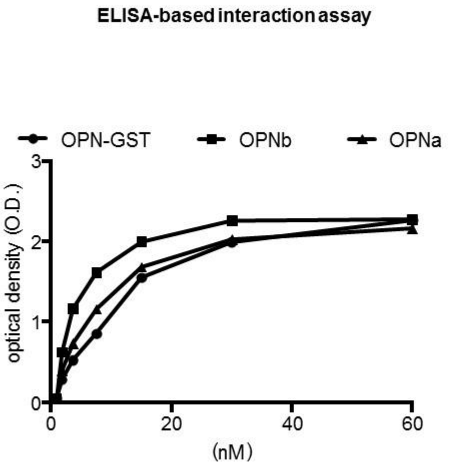

Supplementary Figure 2. ELISA-based interaction assay. The graph shows the interaction of titrated amounts of soluble ICOSL-Fc with a fixed amount of OPN-GST, OPN isoform-a and OPN isoform-b coated on the plate. (black circle) shows OPN-GST, (black square) OPN-b and (black triangle) OPN-a. Data are expressed as means ± standard error (n=3 technical replicate).

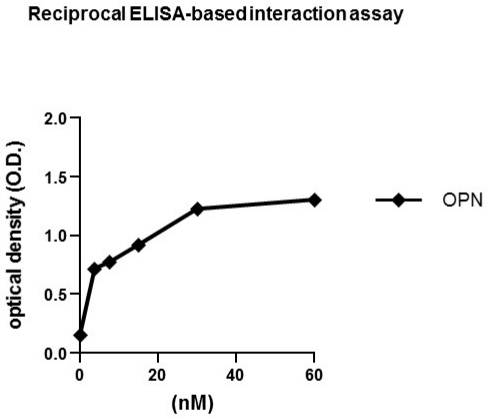

Supplementary Figure 3. Reciprocal ELISA-based interaction assay. The graph shows the interaction of titrated amount of OPN with a fixed amount of ICOSL-Fc (black square). Data are expressed as means ± standard error (n=3 technical replicate).

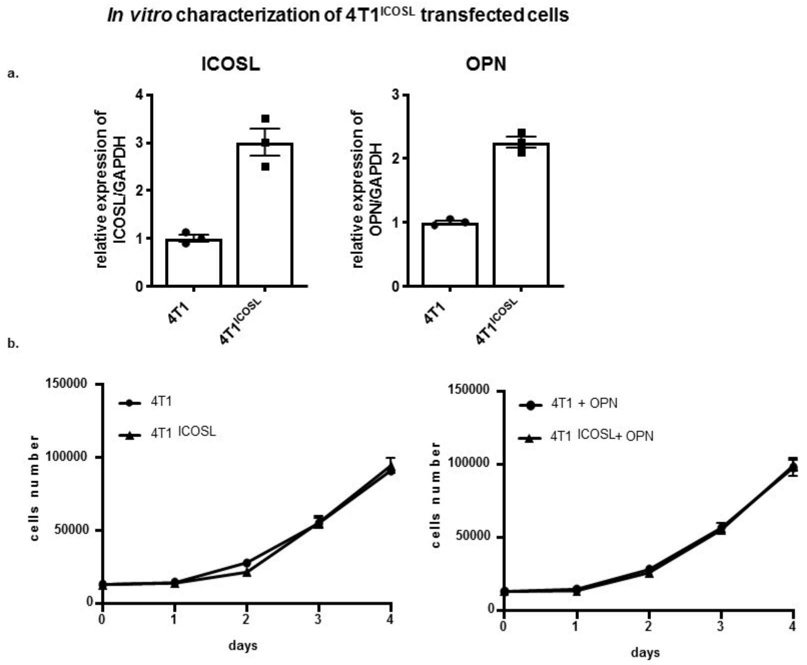

Supplementary Figure 4. ***In vitro*** **characterization of 4T1^{ICOSL} transfected cells. a** Relative quantification of ICOSL and OPN gene expression assessed by real-time PCR in 4T1 cell line and upon ICOSL transfection (4T1^{ICOSL}). Data are shown as the mean gene expression relative to the expression of the endogenous control GAPDH (2−ΔCt method) (n=3 technical replicate). The dot plot represents one of two independent experiments. **b** Kinetic growth assay of 4T1 and 4T1^{ICOSL} transfected cells in the presence and absence of OPN (n=4 technical replicate), the dot plot represents one of two independent experiments; cell counts were determined after trypan blue staining. Data are expressed as means ± standard error.

Body weight of mice injected with 4t1 and 4T1^{ICOSL} cells

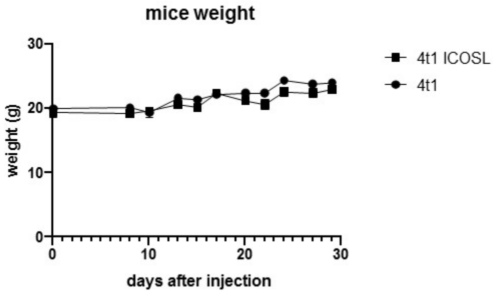

Supplementary Figure 5. **Body weight of mice injected with 4T1 and 4T1^{ICOSL} cells.** Data are shown as mean ± standard error (n=5 biological replicate per group)

Uncropped blots and gels

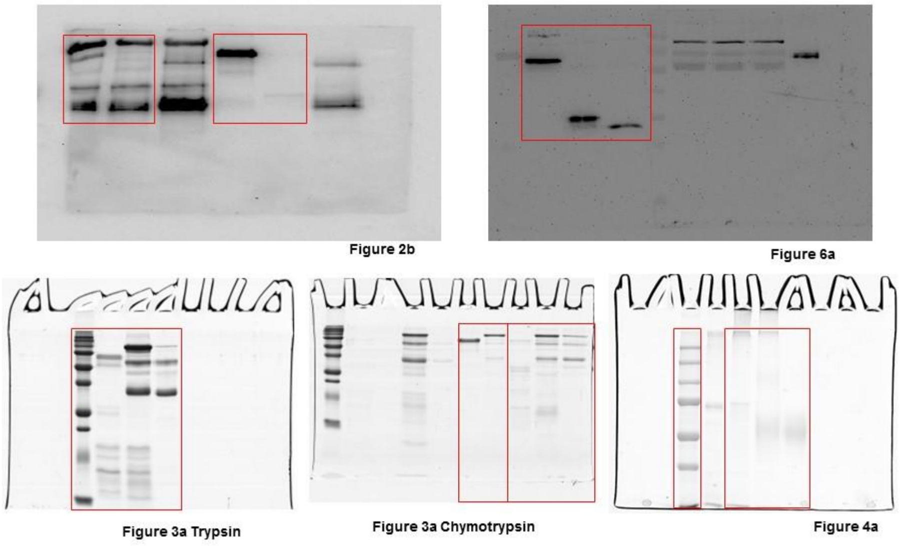

Supplementary Figure 6. Uncropped blot used in figure 2b, 3a, 4a and 6a.
