# Supplementary material for: Osteopontin binds ICOSL promoting tumor metastasis
Source: Commun Biol. 2020 Oct 26;3:615. doi: 10.1038/s42003-020-01333-1 (PMC7588454; doi:10.1038/s42003-020-01333-1)
Supplement: Supplementary file 3 — Description of Additional Supplementary Items [file 42003_2020_1333_MOESM3_ESM.pdf]

Description of additional supplementary file

File name: Supplementary Data 1

Description: Source data underlying the graphs presented in the main and supplementary figures.
