# Supplementary material for: Osteopontin binds ICOSL promoting tumor metastasis
Source: Commun Biol. 2020 Oct 26;3:615. doi: 10.1038/s42003-020-01333-1 (PMC7588454; doi:10.1038/s42003-020-01333-1)
Supplement: Supplementary file 5 — Reporting Summary [file 42003_2020_1333_MOESM5_ESM.pdf]

nature research

Corresponding author(s): Giuseppe Cappellano

Last updated by author(s): Aug 19, 2020

Reporting Summary

Nature Research wishes to improve the reproducibility of the work that we publish. This form provides structure for consistency and transparency in reporting. For further information on Nature Research policies, see Authors & Referees and the Editorial Policy Checklist.

Statistics

For all statistical analyses, confirm that the following items are present in the figure legend, table legend, main text, or Methods section.

n/a	Confirmed	
☐	☒	The exact sample size (*n*) for each experimental group/condition, given as a discrete number and unit of measurement
☐	☒	A statement on whether measurements were taken from distinct samples or whether the same sample was measured repeatedly
☐	☒	The statistical test(s) used AND whether they are one- or two-sided<br>*Only common tests should be described solely by name; describe more complex techniques in the Methods section.*
☐	☒	A description of all covariates tested
☐	☒	A description of any assumptions or corrections, such as tests of normality and adjustment for multiple comparisons
☐	☒	A full description of the statistical parameters including central tendency (e.g. means) or other basic estimates (e.g. regression coefficient) AND variation (e.g. standard deviation) or associated estimates of uncertainty (e.g. confidence intervals)
☒	☐	For null hypothesis testing, the test statistic (e.g. *F*, *t*, *r*) with confidence intervals, effect sizes, degrees of freedom and *P* value noted<br>*Give P values as exact values whenever suitable.*
☒	☐	For Bayesian analysis, information on the choice of priors and Markov chain Monte Carlo settings
☒	☐	For hierarchical and complex designs, identification of the appropriate level for tests and full reporting of outcomes
☒	☐	Estimates of effect sizes (e.g. Cohen's *d*, Pearson's *r*), indicating how they were calculated

Our web collection on statistics for biologists contains articles on many of the points above.

Software and code

Policy information about availability of computer code

Data collection	no softwares were used for data collection
Data analysis	The following software were used for data analysis:<br>GraphPad Instat software (GraphPad Software) was used to statistical analysis,<br>ImageJ software was used to count the tumor colonies and to quantify the Meca-32 positive structures.<br>Batch-Tag, MS-Bridge and MS-Product tools within the Protein Prospector package were used for mass spectrometry analysis in order to identify cross-linked peptide.<br>Peptides after cross-linking experiment were identified using MASCOT software.

For manuscripts utilizing custom algorithms or software that are central to the research but not yet described in published literature, software must be made available to editors/reviewers. We strongly encourage code deposition in a community repository (e.g. GitHub). See the Nature Research guidelines for submitting code & software for further information.

Data

Policy information about availability of data

All manuscripts must include a data availability statement. This statement should provide the following information, where applicable:

- Accession codes, unique identifiers, or web links for publicly available datasets
- A list of figures that have associated raw data
- A description of any restrictions on data availability

The authors declare that the data supporting the findings of this study are available within the article, its supplementary information, and upon request.

Field-specific reporting

Please select the one below that is the best fit for your research. If you are not sure, read the appropriate sections before making your selection.

☒ Life sciences ☐ Behavioural & social sciences ☐ Ecological, evolutionary & environmental sciences

For a reference copy of the document with all sections, see nature.com/documents/nr-reporting-summary-flat.pdf

Life sciences study design

All studies must disclose on these points even when the disclosure is negative.

Sample size	Sample size was based on our preliminary results and, for untransfected 4T1 cells, previous published studies. We used G*power for an alpha=0.05 and beta =0.8.
Data exclusions	No data were excluded from the analysis
Replication	All attempts at replication were succesful.
Randomization	For the animal study, all animals within the two experimental groups were delivered within the same week and derived from the same breeding cage.
Blinding	For the animal study the investigator who mesured tumor growth and metastasis count was blind to the assignment of each experimental group

Reporting for specific materials, systems and methods

We require information from authors about some types of materials, experimental systems and methods used in many studies. Here, indicate whether each material, system or method listed is relevant to your study. If you are not sure if a list item applies to your research, read the appropriate section before selecting a response.

Materials & experimental systems

n/a	Involved in the study	
☐	☒	Antibodies
☐	☒	Eukaryotic cell lines
☒	☐	Palaeontology
☐	☒	Animals and other organisms
☒	☐	Human research participants
☒	☐	Clinical data

Methods

n/a	Involved in the study	
☒	☐	ChIP-seq
☐	☒	Flow cytometry
☒	☐	MRI-based neuroimaging

Antibodies

Antibodies used

The following antibodies were used in this study:
1. Anti-human ICOSL-PE conjugated (Bio-techne, Minneapolis, Minnesota, USA, catalog # FAB165P, clone # 136726, lot AALC0416121)
2. Anti-mouse ICOSL (Life Technologies, Carlsbad, California, USA, catalog # PA5-47161, lot UJ28576)
3. Anti-human HRP conjugated (Dako, Santa Clara, California, USA, catalog # P0214, lot 0005209)
4. Anti-human OPN biotinylated (Bio-techne, Minneapolis, Minnesota, USA, catalog # BAF1433, lot JCY1117031)
5. Anti-human OPN (Bio-techne system, Minneapolis, Minnesota, USA, catalog # AF1433, lot IWX1617021).
6. Anti-mouse OPN for immunofluorescence (Abcam, Cambridge, UK, catalog # ab8448, lot GR3271)
7. Anti-mouse panendothelial (BD, Pharmigen, catalog # 550563, clone Meca-32, lot 5345978)
8. Anti-goat Alexa 555 conjugated (Life technologies, Carlsbad, California, USA, catalog # A21422, lot 1818686)
9. Anti-rat Alexa 488 conjugated (Life technologies, Carlsbad, California, USA, catalog # R37118, lot 1900239)
10. Anti-rabbit Alexa 488 conjugated (Life technologies, Carlsbad, California, USA, catalog # A21206, lot 1927937)

Validation

1. It Detects human B7-H2 in direct ELISAs. In direct ELISAs and Western blots, no cross-reactivity with recombinant human (rh) B7-1, rhB7-2, rhB7-H1, rhB7-H3, or recombinant mouse B7-H2 is observed. It has flow cytometry applications followed by this citation:
B7h triggering inhibits the migration of tumor cell lines. Authors: Dianzani C, Minelli R, Gigliotti C, Occhipinti S, Giovarelli M, Conti L, Boggio E, Shivakumar Y, Baldanzi G, Malacarne V, Orilieri E, Cappellano G, Fantozzi R, Sblattero D, Yagi J, Rojo J, Chiocchetti A, Dianzani U. J Immunol, 2014;192(10):4921-31.

2. In direct ELISAs and Western blots, it has less than 2% cross-reactivity with recombinant human (rh) B7-H2, recombinant mouse (rm) B7-1, rhB7-1, and rmB7-2 is observed. The antibody ID in the antibody register is AB_2577162. It has immunohistochemistry, immunofluorescence and western blots applications.

3. The polyclonal rabbit anti-human IgG/HRP antibody is used to detect human IgG in ELISA assay and immunoblotting, it has the following citations: 1- Vittinghus E. Preanalytical handling of stored urine samples, and measurement of 2-microglobulin, orosomucoid, albumin, transferrin and immunoglobulin G in urine by enzyme-linked immunosorbent assays (ELISA). Scand J Clin Lab Invest 1990;50:843-9. 2- Condorelli F, Scalia G, Stivala A, Gallo R, Marino A, Battaglini CM, et al. Detection of immunoglobulin to measles virus, rubella virus, and mumps virus in serum samples and in microquantities of wholde blood dried on filter paper. J Virol ethods 1994;49:25-36.
4. It Detects human Osteopontin/OPN in ELISAs and Western blots. In sandwich ELISAs, less than 0.2% cross-reactivity with recombinant mouse (rm) Osteopontin and bovine Osteopontin is observed. It has the following applications: western blot, immunohistochemistry and ELISA assay followed by these citations: 1- A multiplex immunoassay of serum biomarkers for the detection of uveal melanoma Authors: J Song, SL Merbs, LJ Sokoll, DW Chan, Z Zhang
Clin Proteomics, 2019;16(0):10. 2- Gene expression signatures for tumor progression, tumor subtype, and tumor thickness in laser-microdissected melanoma tissues. Authors: Jaeger J, Koczan D, Thiesen HJ, Ibrahim SM, Gross G, Spang R, Kunz M Clin. Cancer Res., 2007;13(3):806-15.
5. It Detects human Osteopontin/OPN in direct ELISAs and Western blots. In direct ELISAs, less than 10% cross-reactivity with recombinant rat Osteopontin and recombinant mouse OPN is observed. It is used to recognize human OPN in ELISA assay, immunohistochemistry and also to neutralize OPN in functional experiments. The antibody it is cited from over 15 sources.
6. It is oproduced using the synthetic peptide corresponding to Human Osteopontin aa 170-183 conjugated to keyhole limpet haemocyanin. Sequence: CKSKKFRRPDIQYPD. Its applications are immunohistochemistry, immunofluorescence and western blots. It is cited from over 150 sources.
7. It reacts only with mouse protein. It is recommended to test for immunohistochemical staining and flow cytometry assays. It has up to 5 references.
8. The sensitivity and specificity of each lot is confirmed using ELISA. Minimal cross-reactivity with mouse, rat, human, bovine, guinea pig, and donkey IgG are observed. It is recommended for immunohistochemistry and immunofluorescence assays.
9. This antibody shows minimum cross-reactivity to bovine, chicken, goat, guinea pig, hamster, horse, human, mouse, rat, and sheep serum proteins. It has immunohistochemistry, immunofluorescence and flow cytometry applications, it is cited from over 10 sources.
10. The sensitivity and specificity of each lot is confirmed using ELISA. Minimal cross-reactivity with mouse, rat, human, bovine, guinea pig and donkey IgG are observed. It can be used in flow cytometry, immunohistochemistry and immunofluorescence assays. It is cited from over 5 sources.

Eukaryotic cell lines

Policy information about cell lines

Cell line source(s)	All cell lines used in this study were purchased from ATCC
Authentication	All cell lines used in this study were not authenticated
Mycoplasma contamination	All cell lines used in this study were tested and found negative for Mycoplasma contamination
Commonly misidentified lines (See ICLAC register)	No commonly misidentified cell lines were used

Animals and other organisms

Policy information about studies involving animals; ARRIVE guidelines recommended for reporting animal research

Laboratory animals	Eight-10 weeks old female C57BL/6J, B6.129S6(Cg)-Spp1tm1Blh/J and BALB/cOlaHsd mice<br>Ten-12 weeks old female MRL/MpJ-Faslpr/J mice
Wild animals	This study does not involve wild animals
Field-collected samples	This study does not involve sample collected from field
Ethics oversight	All experimental procedures were conducted, following European guidelines, in accordance with both the University Ethical Committee and the National Institutes of Health Ministry and Care Committee, both of which approved the protocol(DB064.42).

Note that full information on the approval of the study protocol must also be provided in the manuscript.

Flow Cytometry

Plots

Confirm that:

☒ The axis labels state the marker and fluorochrome used (e.g. CD4-FITC).

☒ The axis scales are clearly visible. Include numbers along axes only for bottom left plot of group (a 'group' is an analysis of identical markers).

☐ All plots are contour plots with outliers or pseudocolor plots.

☐ A numerical value for number of cells or percentage (with statistics) is provided.

Methodology

Sample preparation	Hela, A2058, M14, JR8 and PCF-2 cells were dettached and washed twice with PBS + 1% FBS, 2.5 ug of anti-ICOSL-PE or anti-His-PE were used for immunostaning.
Instrument	FACScalibur
Software	Cell quest PRO
Cell population abundance	no sorting experiments were performed
Gating strategy	Hela, A2058, M14, JR8 and PCF-2 cells were identified in FSC/SSC dot plots, then, the PE fluorescence was evaluated.

☐ Tick this box to confirm that a figure exemplifying the gating strategy is provided in the Supplementary Information.
